# Research on the Structure and Properties of Traditional Handmade Bamboo Paper During the Aging Process

**DOI:** 10.3390/molecules29235741

**Published:** 2024-12-05

**Authors:** Zirui Zhu, Kai Zhang, Yu Xue, Zhongming Liu, Yujie Wang, Yanli Zhang, Peng Liu, Xingxiang Ji

**Affiliations:** 1 State Key Laboratory of Biobased Material and Green Papermaking, Qilu University of Technology, Shandong Academy of Sciences, Jinan 250353, China; 2 Institute for Preservation and Conservation of Chinese Ancient Books, Fudan University Library, Fudan University, Shanghai 200433, China; 3 Montverde Academy Shanghai, Shanghai 201318, China; 4 School of Chemistry and Chemical Engineering, Shanghai University of Engineering Science, Shanghai 201620, China

**Keywords:** handmade paper, critical DP, H-bond, hornification

## Abstract

Handmade papers, as carriers of paper-based cultural relics, have played a crucial role in the development of human culture, knowledge, and civilization. Understanding the intricate relationship between the structural properties and degradation mechanisms of handmade papers is essential for the conservation of historical documents. In this work, an artificial dry-heat-accelerated aging method was used to investigate the interplay among the mechanical properties of paper, the degree of polymerization (DP) of cellulose, the chemical composition, the hydrogen bond strength, the crystallinity, and the degree of hornification for paper fibers. The results demonstrated for the first time that the mechanical properties of handmade bamboo paper exhibited an initial plateau region, a rapid decline region, and sometimes a second plateau region as it undergoes a dry-heat aging process. The changes in cellulose, hemicellulose, and lignin content were tracked throughout these three stages. The lignin content was relatively stable, while the cellulose and hemicellulose content decreased, which was consistent with the observed decline in mechanical properties. When the DP of cellulose decreased to the range of 600–400, there was a critical point in the mechanical properties of the paper, marking a transition from the initial stable region to a rapid decline region. The fiber embrittlement caused by cellulose chain breakage resulting from the decrease in DP was counteracted by the enhancement of intermolecular hydrogen bonds and the hornification process. A second stable region appeared when the DP was less than 400, marking a transition from a balanced or slightly decreasing trend in the initial plateau region to a sharp decline. This study also discussed for the first time that the formation of the second plateau region may be due to the presence of hemicellulose and lignin, which hinder the further aggregation of cellulose and maintain the structural stability of the fiber cell. The findings of this study can provide guidance for improving ancient book preservation strategies. On the one hand, understanding how these components affect the durability of paper can help us better predict and slow down the aging of ancient books. On the other hand, specific chemical treatment methods can be designed to stabilize these components and reduce their degradation rate under adverse environmental conditions.

## 1. Introduction

The saying “Paper lasts for a thousand years, while silk endures for eight hundred” highlights the significance of paper as a crucial medium for the transmission and development of human civilization [1]. Traditional Chinese paper, with a history spanning over 2000 years, has played an irreplaceable role in ancient book printing, calligraphy, painting arts, and other cultural relics [2]. The general manufacturing processes for traditional Chinese paper involve steeping, fermenting, washing, steaming, boiling, natural bleaching, pulping, sheet forming, pressing, and drying, employing mild treatment conditions to minimize adverse effects on plant fibers [2,3]. Papers manufactured throughout the long history of Chinese papermaking can be categorized into bast paper, bamboo paper, straw paper, and mixed-fiber paper (i.e., *Xuan* paper), and each is endowed with distinct characteristics [4]. The methods of making various types of paper differ due to the composition and properties of the raw materials, although the basic principles of pulping and papermaking are the same. Longer bast fibers give bast paper better strength, while the mixed paper is a blend of long bast fibers and short rice straw fibers, resulting in excellent effects for calligraphy and painting. Due to its high content of lignin and hemicellulose, bamboo raw material is used to produce a unique type of paper known as bamboo paper. Handmade bamboo paper holds great importance in traditional papermaking in China, with a rich history and a wide variety of categories. The craftsmanship of bamboo paper flourished during the Tang and Song dynasties, particularly in the Song dynasty, where it gained dominance due to its cost-effectiveness, favorable texture, and availability. Furthermore, bamboo paper’s desirable properties, such as its flexibility and water absorption, made it popular in calligraphy and printing. The manual production of bamboo paper reached its peak during the Ming and Qing periods when it was utilized not only for daily writing but also extensively in the restoration and printing of ancient books, as well as in calligraphy and mounting. In 2006, the bamboo-paper-making process was recognized in the first batch of national intangible cultural heritage in China [5]. A survey indicates that some regions in Fujian, Jiangxi, and Zhejiang provinces still produce bamboo paper suitable for archival and ancient book restoration. However, there are also problems, such as the lack of emphasis on bamboo paper, a decline in its quality, a disconnect between production, supply, and marketing, and a lack of successors [6]. More importantly, the relatively short fibers and lower cellulose content of bamboo paper make it less durable than papers made from other materials, such as bast, making it more susceptible to degradation and necessitating specialized conservation and restoration efforts in the restoration and printing of ancient books [1]. Therefore, studying bamboo paper’s aging behavior and preservation methods is crucial for prolonging the lifespan of ancient books and manuscripts and gaining insights into their conservation and restoration needs.

During the long-term preservation process, paper can degrade due to a combination of internal and external factors. Internal factors include acidic degradation products and excessive alkali reserves within the paper itself, while external factors consist of light, temperature, humidity, air pollutants in the environment, and the presence of inks, pigments, fillers, insects, and microorganisms on the paper [7,8,9,10]. To study the degradation of paper, accelerated aging experiments are always conducted in laboratories due to the slow degradation rate under natural conditions [11]. These experiments involve severe conditions, such as elevated temperatures and humidity levels, intense ultraviolet and visible radiation, and significant pollutant concentrations, which expedite the deterioration of paper. Additionally, environmental factors such as high levels of air pollution and adverse meteorological conditions have been shown to exacerbate the degradation process of materials, including paper [12]. In order to gain a deeper understanding of the mechanism behind paper deterioration, researchers often use pure cotton/cotton linter cellulose paper and bleached sulfite softwood/hardwood cellulose paper as model papers [13,14,15]. These model papers are composed predominantly of cellulose, with minimal hemicellulose and lignin content; however, traditional bamboo papers actually contain relatively higher amounts of hemicellulose and lignin, which may exhibit certain differences in aging behavior due to their distinct structural and functional properties.

Researchers have conducted studies on bamboo paper from various perspectives. Compared with the center of the paper pages, the edges of traditional Chinese bamboo paper pages undergo chemical changes through oxidation and photo-aging effects [16,17]. Aging experiments conducted for 72 h at 105 °C in nitrogen, air, and sealed preservation environments reveal that nitrogen storage exhibits the best anti-aging properties, followed by air storage, while sealed preservation performs the worst. Sealed storage is not ideal as it inhibits the release of the paper’s volatile substances. Therefore, it is recommended to use storage equipment that is breathable to allow for air circulation while protecting documents [18].

In their research on bamboo paper aging, Chen and Ding found that handmade bamboo paper with minimal processing is more susceptible to yellowing, while excessive treatment can harm the fibers and impact the thermal stability of the paper [19]. They also developed a quantitative model based on changes in characteristic temperatures of pyrolysis to better evaluate the degree of bamboo paper aging [20]. Additionally, the pyrolysis characteristics of bamboo paper under various dry-heat-aging conditions were studied using thermogravimetric analysis, revealing a deterioration in the thermal stability of bamboo paper. The difference in characteristic temperatures of pyrolysis of bamboo paper, ΔT0.5, was proposed as a parameter to evaluate the degree of bamboo paper aging, with an exponential relationship being established between ΔT0.5 and the retention rate of the tensile index, leading to the development of a quantitative model for assessing bamboo paper aging [21].

A comparative analysis of uncooked and cooked bamboo paper focused on their dimensional stability- and durability-related physicochemical indicators [22]. This study revealed that while uncooked bamboo paper exhibited better dimensional stability, cooked bamboo paper demonstrated superior durability, making it more suitable for meeting the quality requirements of paper used in the restoration of ancient books. Samples of uncooked and cooked bamboo papers were obtained from three paper workshops located in the *Jiangle*, *Liancheng*, and *Changting* regions of Fujian province. The results indicated that although uncooked paper displayed improved dimensional stability, its durability was inferior to that of cooked paper, thus rendering uncooked paper a more suitable material for the restoration of ancient books.

Recently, scholars compared six types of traditional handmade Chinese paper, including bast paper, bamboo paper, and grass paper, and they analyzed their wet-heat-accelerated aging behavior and degradation mechanisms [1]. This study found that the type of raw fiber material directly affects the durability of handmade paper. At the molecular level, most degradation occurs in the non-crystalline regions of cellulose, leading to the breakage of glycosidic bonds, the generation of oxidative groups, and changes in hydrogen bond arrangements in cellulose. Bark paper, due to its specific molecular and supramolecular structure of cellulose, has a longer lifespan and better durability than bamboo paper and grass paper. At the supramolecular level, the cellulose in bast paper has higher crystallinity, providing better stability during the aging process. These results provide valuable information for understanding the degradation mechanisms of handmade paper from different fiber materials and provide a scientific basis for improving the durability of handmade paper. In addition, researchers have conducted accelerated aging experiments under different conditions (humidity or dryness, air or nitrogen) and different durations (2, 4, 10, 25, and 50 weeks) to simulate the chemical degradation of paper [23]. Further research has been conducted on the relationship between chemical degradation and mechanical degradation during the aging process of paper, especially the impact of fibers and fiber-to-fiber bonds on the embrittlement of paper. This emphasizes that the embrittlement of fibers themselves has a greater impact on the overall embrittlement of paper than the degradation of fiber-to-fiber bonds, and the critical threshold of cellulose’s degree of polymerization (DP), below which the embrittlement of paper will significantly accelerate, was pointed out. These findings are of great significance for understanding and predicting issues with the durability and preservation of paper. However, they focus on pure cellulose as the research object, while ancient paper contains a certain amount of hemicellulose and lignin, which have a profound influence on the aging behavior of paper and should also be given attention.

Despite the significance of Chinese handmade papers, the understanding of the relationship between their structure and properties during the aging and degradation process is still limited. The variety of raw materials used in the Chinese papermaking industry, as well as the intricate craftsmanship employed, makes this issue even more complicated. Handmade paper made in traditional crafts often contains a certain amount of lignin and hemicellulose, which, in addition to cellulose, may affect the degradation behavior of the paper. The influence of fiber raw materials on the aging behavior of paper under wet-heat-accelerated aging conditions was investigated. It was found that the longer fiber lengths result in a larger length-to-diameter ratio and a reduced presence of fine fibers in the paper. This, in turn, leads to a higher DP and crystallinity in the original cellulose. As a result, the paper has a longer lifespan and improved durability [1]. A recent study showed that environmental factors may exhibit similar trends in their impacts on the aging process. For pure cellulose paper, mechanical performance gradually decreases above the critical polymerization degree (DPc), while below the DPc, mechanical performance deteriorates significantly [23]. However, traditional bamboo paper often contains higher lignin and hemicellulose content, and the impact of these two components on the durability of the paper has been a subject of ongoing debate. This study focuses on traditional bamboo paper derived from bitter bamboo, aiming to investigate the aging behavior of paper with high lignin and hemicellulose content. It was found that the mechanical properties of bamboo paper with high lignin and hemicellulose content exhibit three stages during accelerated aging in dry heat as the DP decreases: a first plateau region, a rapid decline region, and a second plateau region. This study, for the first time, tracked the changes in cellulose, hemicellulose, and lignin content throughout these three stages. It also explored the reasons for the formation of the second plateau region, which may be attributed to the presence of lignin and hemicellulose inhibiting further cellulose aggregation, thus maintaining structural stability. The findings of this research can enhance our comprehension of the relationship between the structure and properties during the aging and degradation process of ancient paper-based books, provide evaluations and guidance for the development of conservation agents for ancient books, and establish a scientific foundation for the production of more durable and long-lasting handmade paper.

## 2. Results and Discussion

### 2.1. Mechanical Properties

Research on the aging and degradation of paper is typically approached from two perspectives. The first perspective involves studying changes in paper properties over time [24,25]. While this approach provides a direct reflection of the changes in mechanical properties, it can be challenging to determine the underlying causes due to the influence of fiber bonding strength and the inherent strength of the fibers themselves. Factors such as the bonding strength and inherent strength of fibers are influenced by a multitude of factors, with the DP of cellulose playing a significant role in the mechanical properties of paper [26]. Other studies have directly examined the relationship between the reduction in cellulose’s DP and the loss of paper’s mechanical properties under various conditions, including temperature, humidity, and acidity [14,27,28]. The loss of mechanical properties serves as an indicator of paper degradation; however, it is not always a linear function of cellulose degradation. As the DP of cellulose decreases with aging, the breaking of molecular chains leads to fiber brittleness, whether through oxidation or hydrolytic degradation mechanisms [23]. Additionally, a DPc for cellulose (DPc~750, Mn) has been identified, beyond which the mechanical properties decrease significantly, irrespective of the type of mechanical testing conducted [24]. This study builds upon new insights into the relationship between the cellulose DP and the mechanical properties of paper.

Compared with machine-made paper, handmade paper exhibits less variation in fiber orientation during the papermaking process. However, it is possible to control the water flow and reduce fiber orientation through the vibration of the paper screen in manual papermaking. Nevertheless, the water flow still influences the orientation of fibers in the longitudinal and transverse directions, corresponding to the wire and bamboo patterns of the paper mold [2]. This indicates that handmade paper also displays anisotropy in the transverse and longitudinal directions. In this study, the mechanical properties in different directions are referred to as the LD (longitudinal direction) and TD (transverse direction).

**Figure 1:**
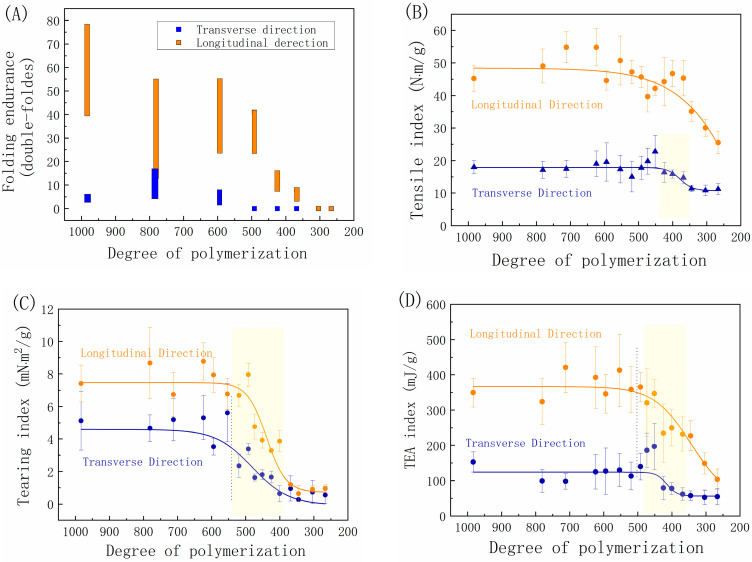
Evolution of mechanical properties (**A**) Folding endurance, (**B**) Tensile index, (**C**) Tearing index and (**D**) TEA index with the decrease in the degree of polymerization (DP) of cellulose, as measured using folding, tearing, and tensile tests on bamboo paper in the longitudinal and transverse directions under an accelerated dry-heating treatment at 105 °C. LD: longitudinal direction; TD: transverse direction. The vertical dotted line indicates the value of the critical DP.

Figure 1 depicts the relationship between the tensile, tear, and folding endurance properties of handmade bamboo paper and its DP. The results reveal several key characteristics. Firstly, unaged handmade bamboo paper is primarily oriented in the longitudinal direction, enabling it to bear and transmit more load in this direction. As a result, it exhibits a higher tensile index and folding endurance than the transverse direction. Due to the ease with which fibers can be pulled out along the paper’s longitudinal direction, while those perpendicular to it hinder this process, the transverse tear index is greater than the longitudinal tear index.

The second characteristic is that the strength properties of the paper exhibit different patterns of decline with a reduction in cellulose DP caused by the extension of the dry-heat-aging time. In the early stages of dry-heat-accelerated aging (Figure 1A–D), the mechanical properties of the paper in the longitudinal direction nearly plateau for a period. However, in the middle and later stages of aging, the tensile index-TD (Figure 1B) and tear index-TD experience a significant decline, followed by a second plateau period (Figure 1C). On the other hand, the tensile index-LD gradually decreases without a second plateau period (Figure 1B). The trend of changes in the energy absorption index is consistent with the tensile index in both the longitudinal and transverse directions (Figure 1D).

Similarly to the phenomena reported in a previous study [23], this work also observed a DPc for the paper. Initially, when the paper’s DP is relatively high, there is a plateau period in its properties. At this stage, cellulose degradation does not necessarily result in a loss of mechanical properties of the paper [29,30]. However, the mechanical properties of the paper decline after surpassing this DPc. The DPc for the paper’s tensile properties and energy absorption index is estimated to be between 450 and 500 (Figure 1B,D), while for tearing performance, it is approximately between 500 and 600 (Figure 1C). Therefore, it can be inferred that the DPc for the plateau and decline periods in this study falls within the range of 450 to 600. This differs slightly from the previously reported Mn-750 [23], which is possibly due to the use of DP obtained from the viscosity-average molecular weight in this study, as well as variations in the raw material content for handmade paper, leading to different degradation processes and, consequently, affecting the DPc.

One consequence of cellulose polymer chain degradation, as the primary component of paper fibers, is an increase in paper brittleness during aging. This property could be assessed through folding endurance [27]. The folding endurance in the longitudinal direction (folding endurance-LD) exhibits a slight decline in the early stages of aging, with the DPc appearing at 500, after which a significant decline is observed (Figure 1A), consistently with previous findings [24]. On the other hand, the change in folding endurance in the transverse direction (folding endurance-TD) is not significant, remaining below 10 overall and dropping to 0 once the DP falls below 600. The folding endurance-TD is lower than 10 due to the paper’s thinness and weaker transverse binding force, coupled with the decrease in fiber strength. In the longitudinal direction, where fibers have certain bonds, the folding endurance-LD gradually decreases along with the weakening of fiber strength. It sharply declines once the DP exceeds 500 and completely disappears when the DP falls below 350. The decline in folding endurance is mainly influenced by fiber brittleness caused by oxidation and cross-linking in the initial stage of aging [26].

### 2.2. DP and Component Contents

**Figure 2:**
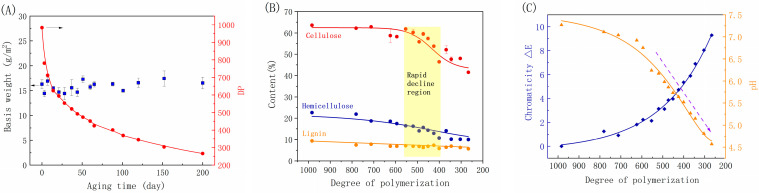
Variations in basic weight and DP as a function of aging time for bamboo paper (**A**); variations in chemical composition (**B**) and chromaticity ΔE and pH (**C**) with the decrease in DP under an accelerated dry-heating treatment at 105 °C.

The DP is a crucial factor in evaluating the performance and longevity of paper [1,31,32]. Accelerated dry-heat aging can significantly decrease the DP of cellulose in paper, a process that is typically divided into three stages (Figure 2A). The initial stage occurs within 30 days, during which the DP of the paper decreases by nearly 40%. Subsequently, as the aging progresses to 50 days, the DP further drops by 10% to around 500. Although the DP continued to decline in this phase, the rate slowed down significantly. The sharp decline in DP results in extensive breakage of cellulose molecular chains, a decrease in fiber strength, and a notable reduction in folding endurance. However, it may not impact the bonding strength between fibers, thus maintaining tensile and tear strength, corresponding to the first plateau region. From 50 days onwards, as the paper continues aging up to 200 days, the DP decreases at a slower rate. Research has indicated that cotton fibers take approximately 850 days for the DP to decrease from 300 to 280, suggesting a slight decrease in molecular weight in the later stages of cellulose degradation [24]. Throughout the aging process from 50 to 200 days, there is a significant decline in the mechanical properties of the paper but a slow degradation of cellulose.

It is often overlooked that during the aging process, the composition of the main components of paper, namely, cellulose, hemicellulose, and lignin, undergo constant changes. As depicted in Figure 2B, the lignin content shows a gradual slight downward trend, particularly in the later stages of aging, where the content remains relatively stable when the DP is below 600. It is important to note that the cellulose and hemicellulose content exhibits a similar trend to that of the mechanical properties when comparing Figure 1 with Figure 2. In Figure 2B, a clear plateau region is evident for the cellulose and hemicellulose content before the DP exceeds 600, with a slight downward trend. Between DP of 600 and 400, a significant decrease is observed. Specifically, the cellulose content decreases from 60% to 50%, and the hemicellulose content drops from 18% to 12%. Once the DP falls below 400, the cellulose decrease rate slows down, while hemicellulose shows a plateau. Additionally, the paper’s pH value gradually declines from an initial 7.25 to 6.8 when the DP is above 600. Subsequently, as the cellulose DP and content decrease, the pH value exhibits a linear downward trend (Figure 2C). Figure 2B illustrates that cellulose and hemicellulose are the main components undergoing degradation, leading to an increase in acidic degradation products. This acceleration of the acidification may be caused by the action of volatile organic compounds on cellulose or hemicellulose self-oxidation. During natural aging, paper undergoes color changes and becomes brittle, which is primarily due to the degradation of cellulose, the primary component of paper fibers [26]. Although the lignin content does not decrease significantly, it may play a role in these processes through changes in its functional groups. Understanding how the slowly changing cellulose, hemicellulose, and lignin content maintains mechanical stability when the DP exceeds 600, i.e., the first plateau region, warrants further investigation.

However, there is limited research explaining the DPc for cellulose [23]. This polymerization threshold is observed in traditional semi-crystalline polymers [33], ensuring a minimum amorphous phase thickness that influences the plastic deformation mechanism. The complexity of cellulose’s microstructure adds to this issue [23,33]. The appearance of this first plateau period seems uncommon in aging studies of pure cellulose samples but emerges when the DP reaches a certain value [29].

### 2.3. Microstructures

The primary constituent of Chinese handmade paper is cellulose, which is a polymer consisting of linear chains of hundreds to thousands of D-glucose units linked via β-(1,4)-glycosidic bonds [34,35]. The hydroxyl groups present in cellulose participate in numerous intra- and intermolecular hydrogen bonds, resulting in various ordered crystalline arrangements [36]. These hydrogen bonds play a crucial role in the mechanical properties of paper by forming inter-fiber connections through interactions between the hydroxyl (–OH) groups in cellulose molecules [37]. The strength and quantity of hydrogen bonds can impact the interlayer spacing and elastic modulus of paper. Moreover, the presence of hydrogen bonds provides the paper with a self-healing capability, as these bonds can dynamically reform under certain conditions, repairing the microstructure of the paper. Some studies suggest that cellulose degradation at the supramolecular level leads to changes in the intensity of hydrogen bonds and the crystallinity of cellulose macromolecules [38,39].

**Figure 3:**
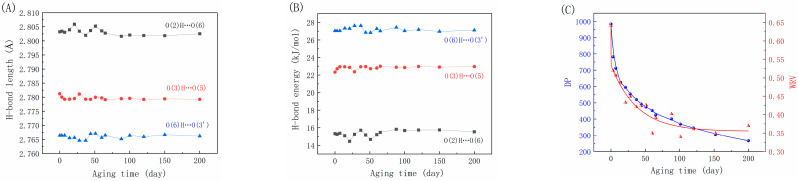
Variations in H-bond length (**A**), H-bond energy (**B**), DP, and water retention value (**C**) as a function of aging time for bamboo paper.

Figure 3 depicts the alterations in hydrogen bond lengths and energies between and within cellulose molecules during the paper-aging process. As shown in Figure 3A,B, both intramolecular and intermolecular hydrogen bond energies increase throughout the aging process, with intermolecular hydrogen bonds displaying a more pronounced change. In the initial 40 days of aging, the bond length of intermolecular hydrogen bonds gradually decreases, indicating a potential movement of cellulose molecular chains toward each other. Subsequently, the bond length increases and stabilizes, fluctuating within a narrow range until day 200. The decrease in intramolecular hydrogen bond length and the increase in bond energy suggest possible contraction or distortion of molecular chains of cellulose due to the prolonged dry-heat treatment. Conversely, the intermolecular hydrogen bonds exhibit a significant decline followed by recovery, suggesting a potential rearrangement process of hydrogen bonds [40] that accompanies the decrease in the paper’s cellulose DP. In the first plateau region of the paper’s mechanical properties, despite the sharp decrease in DP, the increased hydrogen bond energy may strengthen the bonding between fibers, thereby mitigating the decay of the mechanical properties. In other words, while the cellulose molecular chains may experience breakage and make the fibers brittle, the enhanced strength of hydrogen bonds within the internal fiber network of the paper reinforces the binding between fibers and may lead to irreversible hornification.

Hornification refers to the irreversible changes that occur in paper during the water removal process at either room temperature or high temperatures. These changes result in alterations in the paper’s water absorption behavior, including reduced flexibility, decreased water retention capacity, and increased brittleness [41,42]. This phenomenon is attributed to the formation of irreversible hydrogen bonds between microfibrils within the fibers, which are typically associated with the durability and stability of paper. As hornification occurs, the flexibility of the paper decreases. This is because irreversible hydrogen bonds form between the microfibrils within the fibers during the hornification process, which restricts the relative movement of the fibers and reduces the flexibility of the paper. This is due to the breaking of cellulose molecular chains and a decrease in the DP during the aging process, resulting in weakened fiber-to-fiber bonding and increased fragility of the paper [42]. Additionally, hornification leads to a decrease in the water retention value (WRV) of the paper. When preserving and restoring paper-based cultural artifacts, such as ancient books and archives, the WRV is a crucial factor to consider. Papers with a higher WRV are usually more flexible because the moisture between the fibers reduces friction, making the paper easier to bend and fold without tearing. On the other hand, papers with a lower WRV are more prone to brittleness, as the lack of moisture weakens the fiber-to-fiber bonding, making the paper more susceptible to breakage when subjected to external forces. The dimensional stability of paper is also closely related to the WRV, as papers with a high WRV expand when absorbing moisture and contract when drying. A low WRV can lead to brittleness and easy damage to paper, which is crucial for long-term preservation and restoration strategies of cultural artifacts. It is generally believed that the WRV tends to decrease as paper ages, reflecting the degradation and structural changes in cellulose fibers. The findings presented in Figure 3C demonstrate that, similarly to the DP, the change in the WRV can be divided into three stages. However, when DP > 600, there is a clear overall correlation with the WRV, which sharply decreases as the DP decreases. After 50 days of aging, the DP experiences a slow decline, while the WRV exhibits a relatively stable fluctuation trend. Thus, it can be inferred that hornification displays an initial increasing trend followed by a leveling off throughout the entire aging process.

**Figure 4:**
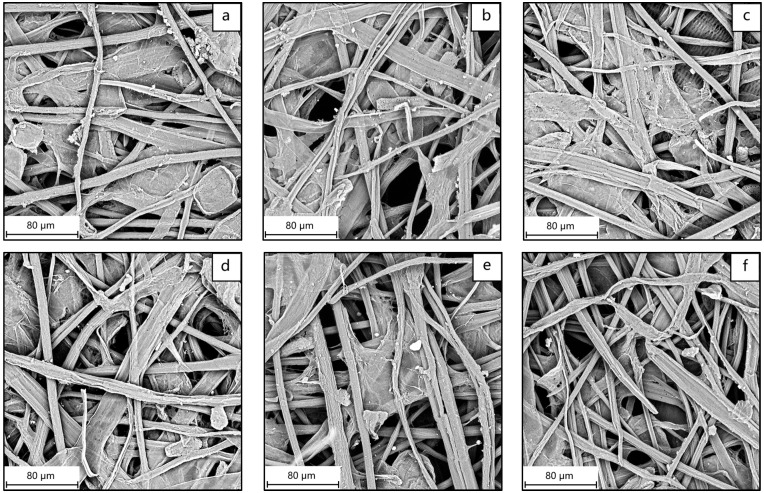
Morphological changes in bamboo paper during accelerated aging: (**a**) 0D, (**b**) 28D, (**c**) 50D, (**d**) 100D, (**e**) 150D, and (**f**) 200D (1000 times).

During dry-heat aging, the fibers gradually shrink from their initial cylindrical shape, with significant collapse and surface wrinkles being observed by day 28 (Figure 4a,b). Subsequently, the shrinkage slows down, but the overall fiber morphology remains stable (Figure 4c–f). Most fiber shrinkage is likely to occur within the first few hours of the drying process [43], which aligns with the trend reflected by the WRV. As the fiber structure contracts during the early stages of aging, the water absorption decreases. Once a certain level of contraction is reached, the fiber morphology remains almost unchanged, while the water absorption performance undergoes irreversible hornification.

**Figure 5:**
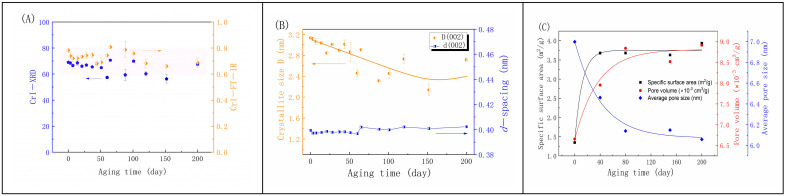
Variations in crystallinity index of cellulose (CrI) derived from XRD and FTIR (**A**), crystallite size D and *d*−spacing (**B**), and pore parameters (**C**) during thermal drying for bamboo paper.

Research has also indicated a correlation between mechanical properties and crystalline structure during paper aging [16,44]. The supramolecular structure of cellulose affects the degradation of its molecular structure, with a higher supramolecular order hindering degradation [39,45]. Although aging causes a reduction in the adsorption and swelling capacity of the paper, leading to an increased crystallinity [41], the infrared OKI crystallinity index shown in Figure 5A demonstrates a significant decrease in the first 3 days, followed by an increase period (3–40 days), and then a decrease after 100 days, eventually leveling off within a certain range. The X-ray diffraction (XRD) results show an initially stable period, followed by regular fluctuations after 50 days. The main interplanar distances, especially for the 002 plane, remain generally stable. However, the crystal grain size gradually decreases from 8 layers to 5–7 layers in the later stages (Figure 5B). This change is likely attributed to the hornification effect caused by cellulose microfibrils during the drying process. In the later stages of paper aging, although thermal aging leads to fiber contraction or collapse, the amorphous regions of cellulose may have degraded significantly. Cellulose co-crystallization, which involves hydrogen bonds, hydrophobic interactions, and van der Waals forces within fibers, was not observed. It may be due to the inhibition of lignin and hemicellulose from the hornification of paper cellulose, preventing the enlargement of crystalline particles and the aggregation of cellulose microfibrils [46]. Moreover, hemicellulose and lignin may contribute to bonding and maintaining the stability of the fiber structure despite the decrease in the cellulose DP. Xylan and glucomannan are important types of hemicellulose that have been found to reduce hornification [43,47].

Figure 5C illustrates the variation in pore parameters in paper fibers during the thermal drying process. In the early stages of aging, the specific surface area rapidly increases and stabilizes after 40 days, indicating that the fiber surface becomes rougher and more porous. The pore volume also shows a similar trend but with a slower growth rate, possibly due to the combined effect of pore size and quantity. In contrast, the average pore diameter gradually decreases as aging progresses, implying a contraction of the internal pore structure of the fibers. As the average pore size decreases while the pore volume increases, this indicates an increase in the number of pores. This may be attributed to the rapid breakage and degradation of cellulose molecular chains in the early stages of aging, leading to the formation of porous fibers. However, after 80 days of aging, the pore volume and average pore diameter of the fibers show a tendency to stabilize, indicating that the pore structure has reached a stable state. The average pore size does not further decrease, suggesting that the fibers maintain a relatively stable structure in the later stages of aging. Combining the previous discussion, this may be due to hemicellulose and lignin hindering cellulose aggregation and co-crystallization, maintaining the stability of the fiber skeleton structure. This phenomenon has also been reported in thermal treatment processes [43].

### 2.4. Aging Mechanisms

**Figure 6:**
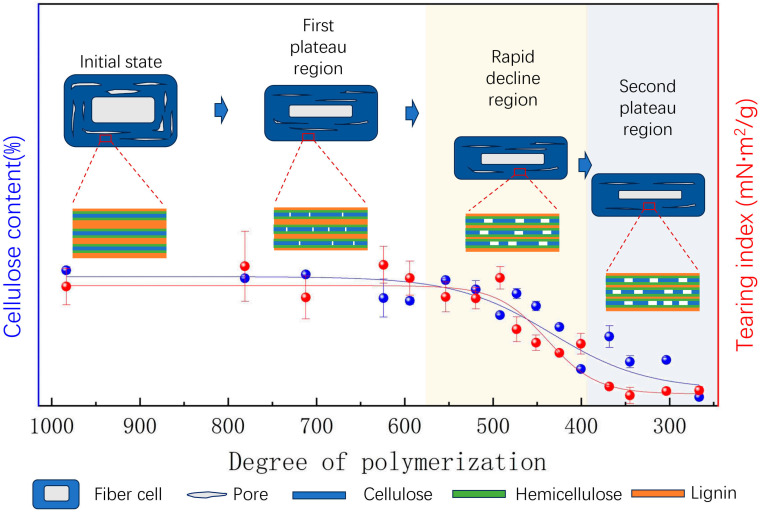
Schematic illustration of the structure and properties of traditional handmade bamboo paper during the aging process.

The aging behavior of bamboo paper is highly complex due to the presence of a certain proportion of hemicellulose and lignin in addition to cellulose in its chemical composition. As mentioned earlier, the mechanical properties exhibit three regions with respect to DP: the first plateau region, the rapid decline region, and the second plateau region (Figure 6). The DP of cellulose significantly impacts the mechanical properties of paper, and there is a similar trend between the cellulose content and the DP. It is evident that oxidative degradation dominates due to the significant decline in DP [23], leading to the speculation that fiber embrittlement may play a more significant role in the first plateau region than the deterioration of fiber–fiber bonds. It is commonly believed that the degradation of cellulose leads to chain breakage, reducing the inter-fiber bonding force and, thereby, decreasing the mechanical properties of paper. However, this study demonstrates that despite a significant decrease in cellulose DP, which would normally lead to brittleness in the initial plateau stage of fiber degradation, this brittleness does not significantly impair the mechanical performance of the paper. This may be attributed to the role of hydrogen bonds, as the strength of paper in the first plateau region remains unchanged. Additionally, the decrease in the WRV indicates the appearance of irreversible bonding in the paper, with some hornification occurring. The rearrangement of hydrogen bonds and hornification may compensate for the impact of cellulose chain breakage on mechanical strength. These hydrogen bonds, which are formed via free hydroxyl groups, create strong bonds within the fibers that are, to some extent, irreversible. The resulting multiple hydrogen bond structures are highly stable and not easily disrupted [48]. Additionally, the formation of ester bridges between cellulose molecular chains, which are covalent bonds and irreversible in water, contributes to hornification [49]. These various mechanisms may all contribute to the hornification process and potentially interact with each other. For instance, functional groups within the cellulose chains may engage in hydrogen bonds, ester bonds, ether bonds, and other types of bonding interactions. It is important to note that hornification is not entirely detrimental. This study suggests that moderate hornification can be strengthened by the hydrogen bonds between cellulose molecules, thereby enhancing the binding between fibers, helping to preserve the mechanical properties of paper, and preventing a sharp decline in the initial stage. It is important to note that the decrease in cellulose DP does not occur simultaneously with the decrease in cellulose content. During the aging process, cellulose chains may break, forming shorter chains, but this does not immediately reduce the total cellulose content. There may also be recombination of chains, keeping the total cellulose content relatively stable for a certain period of time.

Once the cellulose DP falls below a critical value, a region of rapid decline appears. In this region, the most noticeable manifestation is further breakage of cellulose chains and the release of a large number of small molecules, leading to a rapid decrease in cellulose content and causing a series of adverse reactions, such as a decrease in paper acidity and a drastic decline in its mechanical properties. The degradation of cellulose or hemicellulose in paper can also generate volatile components that promote paper acidification. Cellulose in paper contains 1,4-β-glycosidic bonds, which are sensitive to acids. Its breakage leads to a decrease in the mechanical strength of the paper, as the reduction in DP affects the physical structure of the paper and the bonding between fibers. The structure of hemicellulose is more complex than that of cellulose, as it is composed of various sugars, including glucose, mannose, xylose, arabinose, and galactose. Under acidic conditions, hemicellulose also undergoes hydrolysis reactions, causing the glycosidic bonds to break and producing various monosaccharides [10].

The impact of hemicellulose and lignin on the aging behavior of bamboo paper is more positive than expected. Although the decrease in hemicellulose and lignin content is limited throughout the aging process, it is possible that their presence contributes to the appearance of the second plateau region.

Some studies suggest that lignin negatively impacts paper durability [14]. It plays a dual role in the degradation of cellulose—as an oxidation catalyst (source of radicals) and as an antioxidant [14,50]. In its role as an oxidative catalyst in an acidic paper, lignin generates free radicals that initiate and accelerate the oxidation process of cellulose. This process involves the hydroxyl (–OH) groups on the cellulose molecular chains, which can be oxidized into carbonyl (>C=O) and carboxyl (–COOH) groups, among other oxidation byproducts. This not only reduces the mechanical strength of the paper but also leads to changes in its chemical structure [14], further contributing to the yellowing of papers with high lignin content [51]. Recent studies have indicated that the lignin content does not affect the aging rate of paper produced at a neutral pH [52]. This is likely due to the cross-linking between lignin and carbohydrates effectively preventing the formation of hydrogen bonding in the fibers, both internally and externally, and reducing irreversible hornification [53].

The unchanged WRV indicates that fiber hornification does not deepen further, and the crystallinity does not continue to increase in this stage. Additionally, observations of the morphology of paper fibers show that after an initial significant contraction, there is no further contraction. Thus, the presence of hemicellulose and lignin limits the intermolecular interactions between cellulose molecules, slowing down the aggregation of the fiber filaments and maintaining the integrity of the fiber structure in the second plateau region.

This study only discusses the dry-heat aging behavior of handmade bamboo paper containing a certain amount of hemicellulose and lignin. However, different components of paper have their own characteristics in terms of chemical degradation processes under different aging conditions. Only through a comprehensive analysis of the structure and properties of paper under different aging conditions can a comprehensive conclusion of chemical degradation leading to changes in the mechanical properties of paper be provided. The chemical degradation of paper mainly occurs through the hydrolysis and oxidation of cellulose, which leads to embrittlement and failure of the paper [10]. A decrease in the DP reduces the effective mechanical properties of the fibers, ultimately leading to fiber embrittlement and loss of material integrity. In addition, the acidity, moisture content, and microstructural characteristics of the paper also affect its chemical and mechanical degradation behavior. Under different aging conditions, the mechanical behavior of paper varies. For example, high-humidity environments accelerate the hydrolysis of cellulose, causing the paper to embrittle more quickly. Conversely, in dry conditions, although the hydrolysis rate slows down, oxidation may become the primary degradation mechanism. Furthermore, the acidity of the paper affects its degradation rate, as an acidic environment accelerates the hydrolysis of cellulose, thus affecting the mechanical properties of the paper [7]. However, other protective materials may exhibit different aging behaviors from those of paper. For example, some polymer or synthetic materials may have different sensitivities to environmental conditions such as humidity and temperature or exhibit different mechanical behaviors during the aging process. These materials may have better aging resistance or exhibit more stable mechanical properties under specific conditions. However, their limitations lie in the fact that the glass transition temperature or that the melting point of polymers is higher than room temperature but lower than the aging temperature, making it difficult to evaluate their durability in relation to paper and further development of a comprehensive evaluation system is needed.

## 3. Materials and Methods

### 3.1. Materials

The bamboo paper used in this study was made from bitter bamboo and was crafted by hand using traditional craftsmanship. According to the “*Fenghua* City Chronicles”, *Tang’ao* bamboo paper was first recorded in historical books in the ninth year of the *Zhengde* period of the Ming Dynasty (1514 AD), and it has a history of nearly 500 years. It can be used for the restoration of ancient books, and it has a thickness of 0.08 mm. The traditional bamboo papermaking process is roughly as follows: (1) harvesting bamboo for raw material; (2) air-drying the bamboo; (3) splitting the bamboo into strips; (4) soaking the strips in slaked lime; (5) cleaning the bamboo strips; (6) steaming for seven days; (7) pounding the bamboo into a pulp; (8) washing the paper pulp; (9) mixing with kiwi vine juice; (10) using a bamboo mat to form the paper; (11) pressing and drying; (12) removing the paper and trimming the edges. Copper ethylenediamine (CED) solution (Bis(ethylenediamine)copper(II) hydroxide solution (1 M in H_2_O)) was purchased from Sigma-Aldrich (Shanghai) Trading Co., Ltd., Shanghai, China.

### 3.2. Accelerated Aging

The artificial aging treatment of handmade papers followed the Chinese standard GB/T464-2008 [54] (equivalent to ISO 5630-4:1986.MOD [55]) for dry-heat aging with the temperature set to 105 ± 2 °C and circa 0% RH. Sample collection was carried out according to a predetermined number of aging days. Sampling intervals were shorter in the early stages of aging and longer in the later stages. This condition was set to simulate the temperature effect during the natural aging process as closely as possible under controlled laboratory conditions in order to reflect the oxidation, fracture, and degradation of the paper itself.

### 3.3. Analysis of Chemical Components

The standard method of the United States Department of Energy was applied to quantitatively analyze the cellulose, hemicellulose, and lignin in the materials. The types and content of sugars in the hydrolyzed products were determined using high-performance liquid chromatography (HPLC). A 0.3 g sample was placed in a 10 mL crucible and mixed with 3 mL of 72% H_2_SO_4_, allowing it to swell for 60 min. Subsequently, 84 mL of distilled water was added to dilute the H_2_SO_4_ concentration to 4%. The mixture was reacted at 121 °C for 1 h and then filtered to obtain a solid residue (Klason lignin) and a filtrate. The acid-soluble lignin content in the filtrate was measured using Agilent 8453 ultraviolet–visible spectrophotometry (Agilent Technologies, Inc., Santa Clara, CA, USA) at 205 nm. The sugar content in the filtrate was analyzed via HPLC (LC-20AT) with an Aminex HPX-87H column (Bio-Rad Laboratories, Inc., Hercules, CA, USA) under the following conditions: a column temperature of 55 °C, a mobile phase of 5 mmol/L dilute H_2_SO_4_, and a flow rate of 0.6 mL/min.

### 3.4. Viscosity Determination

The DP value of the paper cellulose was measured using the viscosity method according to a report [1]. Paper samples were weighed and added to a plastic bottle with 10 mL of deionized water. After shaking for 30 min, 10 mL of CED (1 M in H_2_O) solution was added. The plastic bottle was shaken evenly for 1 h at 25 °C until the paper specimen was dissolved completely. Then, the obtained solutions were transferred into a capillary viscometer, and the time of solution declining from the top to the bottom was recorded. Due to the high lignin content in the bamboo paper studied here, which was higher than 5%, the standard method for determining the intrinsic viscosity-related molecular weight of cellulose was no longer applicable. In this study, the intrinsic-viscosity-related molecular weight of cellulose was determined using a modified copper ethylenediamine method to determine the intrinsic viscosity and DP of handmade paper [56]. For handmade paper with a lignin content below 10%, the cellulose viscosity and DP can be obtained using the CED method based on Equation (1):

(1) $${\left[\eta \right]}_{c}=\frac{{\left[\eta \right]}_{l}}{1-\chi \%},$$

where [*η*]*_c_* represents the intrinsic viscosity of cellulose without accounting for the lignin content (in mL/g); [*η*]*_l_* is the intrinsic viscosity of the model substance while accounting for lignin content (in mL/g); *χ*% is the mass fraction of lignin.

The Martin empirical equation was used to calculate the DP of the paper samples (Equations (2)–(4)).

(2) $$\eta_{r}=h_{n}*t_{n},$$

(3) $${\left[\eta \right]}_{c}=\eta_{r}/\rho ,$$

and

(4) $${DP}^{0.905}=0.75{\left[\eta \right]}_{c},$$

where $\eta_{r}$ is the relative viscosity of paper cellulose; $h_{n}$ is the constant of the viscosimeter (0.0703 s^−1^); $t_{n}$ is the recorded time (s); ${\left[\eta \right]}_{c}$ is the intrinsic viscosity of paper cellulose; *ρ* is the concentration of the paper solution (g/mL).

### 3.5. Tests of Mechanical Properties

The tensile index of paper was measured according to GB/T 12914-2008 [57] at a constant elongation rate of 20 mm/min using a tensile strength tester (ZB-WLQ, Hangzhou Zhibang Automation Technology Co., Ltd., Hangzhou, China). The folding endurance was measured according to GB/T 475-2008 [58] with an MIT folding endurance tester (ZB-NZ135A, Hangzhou Zhibang Automation Technology Co., Ltd., Hangzhou, China). The tearing index was assessed according to GB/T 455-2002 [59] with a tearing strength tester (ZB-SL, Hangzhou Zhibang Automation Technology Co., Ltd., Hangzhou, China). The properties of the laboratory paper sheets were determined at a temperature of 23 °C and 50% relative humidity. The tensile index of paper is equal to the tensile strength divided by the base weight, and the tearing index is equal to the tear strength divided by the base weight.

### 3.6. Infrared Analysis

The attenuated total reflection Fourier transform infrared (ATR-FTIR) spectrum of the paper sample was determined on a Spectrum Two Spectrometer (PerkinElmer, Inc., Waltham, MA, USA) equipped with a diamond ATR detector. The scan scope was 4000–400 cm^−1^. The original spectra were calibrated to eliminate the effects of radiation wavelength on the intensities of the absorption bands.

The hydrogen bond energy ($E_{H}$) was calculated using Equation (5) [60]:

(5) $$E_{H}=\frac{1}{k}\left[\frac{v_{0}-v}{v_{0}}\right],$$

where 1/*k* = 2.625 × 10^2^ kJ, $v_{0}$ is the frequency of the standard free hydroxyl group (3650 cm^−1^), and v is the calculated frequency of the hydroxyl group.

The hydrogen bond length (*R*) was calculated using the Sederholm equation (Equation (6)):

(6) $$v_{0}-v=4.43\times 10^{3}\;(2.84-R),$$

where $v_{0}$ is the stretching vibration frequency of a single hydroxyl group (3600 cm^−1^), and v is the calculated frequency of the hydroxyl group.

### 3.7. Water Retention Value Measurement

The water retention value of the sample was tested as follows: 2 g of the sample was immersed in distilled water for 3 h, removed, and placed within a WRV tester to centrifuge for 30 min. The weight of the sample post-centrifugation was denoted as *M*_1_. After that, the sample was transferred into an oven and dried at 105 °C for 4 h. After cooling, the weight of the sample, *M*_2_, was measured, and the formula for calculating the water retention value was as follows:

(7) $$WRV=\frac{M_{1}-M_{2}}{M_{2}}.$$

### 3.8. Chromaticity Test

The chromaticity of the paper was determined with an automatic colorimeter. To ensure accuracy and minimize potential errors, each sample was measured six times at different locations. The change in chromaticity ∆*E* was calculated via the following equation:

(8) $$∆E=\sqrt{{∆L}^{2}+{∆a}^{2}+{∆b}^{2}},$$

where *L*, *a*, and *b* represent three different colorimetric coordinate values, respectively. *L* indicates the lightness, a indicates red–green, and b indicates yellow–blue. ∆*L*, ∆*a*, and ∆*b* are the differences between the corresponding values of different samples.

### 3.9. X-Ray Diffraction Measurement

The X-rays from a Cu tube operating at 30 kV and 10 mA were collected with an energy-dispersive detector that was able to resolve the Cu-K*α* line (*λ* = 0.154184 nm). The X-ray source was a copper target bombarded with electrons. Scans were obtained from 5° to 40° 2*θ* using a step size of 0.05°.

The crystallinity index of cellulose was calculated from the XRD spectra using the following equation:

(9) $$CrI=\frac{I_{200}-I_{AM}}{I_{200}}\times 100\%,$$

where *I*_200_ and *I_AM_* are the scattering intensities from the diffraction intensity of the (200) lattice plane and the height of the minimum value between the (200) and the (110) peaks, respectively.

The *d*-spacing was calculated using Bragg’s equation [10], and the crystallite sizes were calculated using the Scherrer equation [11]:

(10) nλ=2dsinθ

and

(11) D=0.9λ/(βsinθ)

where *n* is an integer; *λ* is the incident wavelength; *d* is the spacing between the planes in the atomic lattice; *θ* is the angle between the incident ray and the scattering planes; *D* is the crystallite size perpendicular to the plane; *β* is the full width at half-maximum in radians.

### 3.10. Low-Temperature Nitrogen Absorption

The low-temperature nitrogen adsorption measurement was conducted on the surface area and porosity analyzer (ASAP-2420, Micromeritics, Norcross, GA, USA). The sample (0.3–0.5 g, oven-dried weight) was then degassed at 120 °C for at least 8 h. The N_2_ adsorption/desorption isotherms were analyzed at 77 K, and the specific surface area was calculated using the BET method. The average pore size and the pore volume were calculated using the BJH method.

## 4. Conclusions

This study explores the structural and performance changes in traditional Chinese bamboo paper during the process of degradation through dry-heat aging. The research reveals that the DP of cellulose undergoes three phases: an initial plateau phase, a rapid decline phase, and a second plateau phase. A critical performance threshold is observed when the DP ranges from 600 to 400, marking a shift from a balanced or slightly decreasing trend in the initial plateau phase to a sharp decline. There is a distinct correlation between the decrease in cellulose content in paper and the deterioration of certain paper properties. This study also discusses for the first time that the formation of the second plateau phase may be due to the presence of hemicellulose and lignin, which hinder further aggregation of cellulose and maintain structural stability, providing the paper with some strength even after 200 days of dry-heat aging. While there are limitations in this study, obtaining detailed insights into hydrogen bond rearrangements and chemical group bonding would enhance explanations from a chemical and structural perspective, providing a deeper understanding of the structural and performance changes in the bamboo paper during degradation through dry-heat aging. This research can provide valuable guidance for traditional papermaking practices and the preservation of ancient books. For papers that contain a certain amount of hemicellulose and lignin, these components may play a role in maintaining structural stability during longer-term preservation. Meanwhile, selecting papers with a higher cellulose DP can prolong the duration of the initial plateau phase, thereby extending the preservation time of ancient books. On the other hand, this study provides multidimensional considerations for the development of materials for the preservation of ancient books. After implementation, it is important to examine whether the degradation and hornification of paper are inhibited and whether the maintenance of apparent performance affects the extent of internal degradation. The limitations of this study include the need to systematically compare the degradation behavior of bamboo paper under different humidity conditions, UV exposure, or exposure to different air pollutants and to establish dynamic or thermodynamic models that describe the relationship between cellulose degradation and accelerated aging parameters under the influence of hemicellulose and lignin. This would provide a scientific basis for a deeper understanding of aging mechanisms and the restoration and preservation of traditional ancient books.

## Data Availability

Data are contained within the article.
